# Pretreatment subcutaneous adipose tissue predicts the outcomes of patients with head and neck cancer receiving definitive radiation and chemoradiation in Taiwan

**DOI:** 10.1002/cam4.1365

**Published:** 2018-04-02

**Authors:** Ping Ching Pai, Chi Cheng Chuang, Wen Ching Chuang, Ngan Ming Tsang, Chen Kan Tseng, Kuan Hung Chen, Tzu Chen Yen, Chien Yu Lin, Kai Ping Chang, Kin Fong Lei

**Affiliations:** ^1^ Department of Radiation Oncology Chang Gung Memorial Hospital and University at Lin‐Kou Taoyuan Taiwan; ^2^ Department of Neurosurgery Chang Gung Memorial Hospital and University at Lin‐Kou Taoyuan Taiwan; ^3^ School of Medicine Chang Gung University Taoyuan Taiwan; ^4^ School of Traditional Chinese Medicine Chang Gung University Taoyuan Taiwan; ^5^ Departments of Nuclear Medicine and Molecular Imaging Center Chang Gung Memorial Hospital and University at Lin‐Kou Taoyuan Taiwan; ^6^ Department of Otolaryngology‐Head and Neck Surgery Chang Gung Memorial Hospital and Chang Gung University at Lin‐Kou Taoyuan Taiwan; ^7^ Graduate Institute of Medical Mechatronics Chang Gung University Taoyuan Taiwan

**Keywords:** Body mass index, head and neck cancer, subcutaneous adipose tissue, survival

## Abstract

We aimed to determine whether body composition assessment before treatment can predict outcomes in patients with head and neck cancer (HNC). All 881 patients with locoregional head and neck cancer treated with curative intent radiotherapy (RT) between 2005 and 2012 were retrospectively investigated. Body composition was analyzed via pre‐RT planning computed tomography (CT) images. Subcutaneous adipose tissue (SAT) and skeletal muscle (SM) indices were measured cross‐sectionally at the level of the third thoracic vertebra. Overall survival (OS), locoregional control (LRC), and distant metastasis‐free survival (MFS) were analyzed by body composition index and body mass index (BMI). Survivors were followed up for a median of 4.68 years. The SAT indices in female patients were significantly higher than those in males (*P* < 0.001). The median SAT and muscle indices were 18.6 and 34.3 cm^2^/m^2^ for women and 6.19 and 51.74 cm^2^/m^2^ for men, respectively. The 5‐ and 10‐year MFS, LRC, and OS rates were 83% and 82.1%, 73.4% and 71.4%, and 66.4 and 57.6%, respectively. Higher pretreatment SAT index was associated with MFS (hazard ratio [HR]: 0.65; *P* = 0.015), LRC (HR: 0.758; *P* = 0.047), and OS (HR: 0.604; *P* < 0.001). Higher pretreatment BMI was associated with MFS (HR: 0.642; *P* = 0.031) and OS (HR: 0.615; *P* < 0.001). The pretreatment SM index had no significant effect on MFS, LRC, and OS. Multivariate analysis revealed that T‐stage, N‐stage, lesion sites, age, and RT treatment days are independent factors associated with OS; T‐stage, N‐stage, and lesion sites are independent factors associated with MFS; and N‐stage, smoking history, and betel quid chewing history are independent factors associated with LRC. A higher CT‐assessed SAT index predicts superior MSF, LCR, and OS in patients with curative HNC, whereas SM does not predict survival or locoregional control.

## Introduction

Significant weight loss is common among patients with cancer, especially in pharyngolaryngeal cancers. Body mass index (BMI) is an indicator of nutrition in patients with cancer; its elevation is significantly associated with superior survival in a variety of cancers 1, 2, 3, even in patients with distant metastases (DM) 4. However, BMI is still considered a poor measure of body composition 5, as weight loss does not represent the severity of adipose tissue depletion and muscle wasting 6. The majority of patients with cancer experience varying degrees of muscle wasting and/or fat loss. Potential links between body composition and prognosis in patients with cancer have been identified 7, 8, 9; however, the importance of fat loss during cancer affliction is poorly understood, especially as most investigations have focused on sarcopenia, the loss of lean muscle mass without synchronized loss of fat mass 10. Few studies have focused on the association between fat loss and patients’ outcomes; these have shown that decreased adipose tissue is a poor prognostic indicator in advanced cancer regardless of patients’ weights 9, 11, 12.

Dural‐energy X‐ray absorptiometry (DEXA) has been the standard for evaluating body composition with an advantage of a lower radiation exposure 13; however, DEXA is limited in directly estimating of muscle mass or lean body mass. Currently, computed tomography (CT) and magnetic resonance imaging (MRI) provide a high quality of specificity and accuracy in distinguishing body organs and tissues 14, 15. Methods for measuring body fat include measurements of potassium‐40 content, gas dilutions, and proton activation; densitometry; skinfold thickness; bioelectrical impedance; soft‐tissue radiography; ultrasonography; MRI; and CT 14, 16, 17, 18.

Computed tomography (CT) is ideal for assessing body fat distribution because fat intensity is distinct from that of other tissues 19. Furthermore, CT allows for separate quantification of visceral and subcutaneous fat using multidetection techniques with high‐speed scanning and high spatial resolution.

Sex disparity in both absolute body fat and proportion thereof has been observed 20. Women have substantially more total adipose than men; moreover, women have significantly more subcutaneous adipose tissue while men predominantly accumulate visceral adipose tissue 21, 22. The Surveillance, Epidemiology, and End Results (SEER) database has shown that mortality rate ratios (MMR) are significantly higher among males than females for the most cancers 23. Laryngeal cancer and hypopharyngeal cancer are leading cancers with highest male‐to‐female MRR. A similar phenomenon of sex differences in outcomes of pharyngolaryngeal cancer was also observed in Taiwan 3, while greater exposure to carcinogens and delayed diagnoses of malignant cancer in men may contribute to this phenomenon 24, 25. However, considering sex differences in body composition and mortality rates, higher volumes of fat in women may influence the outcomes of patients with pharyngolaryngeal cancer. Therefore, we conducted a retrospective study to determine whether subcutaneous fat before treatment is associated with outcomes in patients with head and neck cancers (HNCs) in Taiwan who undergo definitive radiotherapy (RT) or chemoradiation (CRT) with curative intent.

## Subjects and Methods

### Patients and clinical treatment

This retrospective study was approved by our Institutional Review Board. We identified 1,957 patients who were diagnosed with HNC and underwent curative RT without primary surgery between March 2005 and February 2012. All patients underwent complete staging evaluation according to the 2012 American Joint Committee on Cancer TNM staging system. The exclusion criteria were patients who were younger than 18 years or had secondary primary cancers within 3 years after treatment, histologically diagnosed sarcoma, DM at the time of diagnosis, RT treatment for longer than 10 weeks, or CT simulation performed more than thirty days before RT. The standard treatment for patients with HNC included definitive RT or CRT with curative intent. The biological equivalent in 2‐Gy equivalents fractions, using the equivalent dose in 2‐Gy fractions (EQD2), was calculated according to the size, and number of fractions was adjusted for various dose‐fractionation programs. The total EQD2 ranged from 64 to 74 Gy for all patients. Ultimately, we identified 1,648 patients treated with curative intent.

Pretreatment body weight was measured within 14 days before RT; pretreatment BMI was calculated as weight in kilograms divided by the square of the height in meters. Chemotherapy was documented from 1 month before RT to the last follow‐up.

### Body composition based on CT imaging assessment

**Figure 1:**
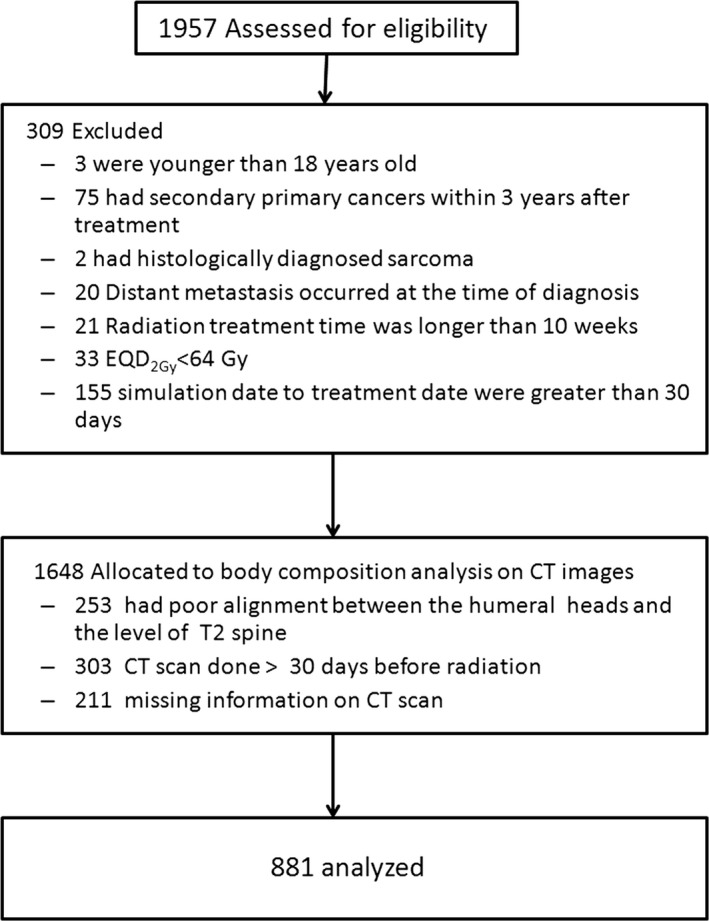
Enrollment flowchart for the study population.

The subcutaneous adipose tissue (SAT) volume and skeletal muscle (SM) mass were evaluated using images obtained during CT simulation for RT treatment planning (Eclipse^TM^ version 8.2, Varian Medical System, Palo Alto, CA, USA). All images were collected within 30 days before RT. CT images acquired during simulation with 3‐mm slice thickness without contrast enhancement were obtained from the vertex‐to‐nipple level for HNCs. A transverse image slice was chosen along an alignment of both humeral heads and the secondary thoracic vertebra (T2) for analysis; images generated during CT simulation for head–neck cancer do not include the abdomen to avoid unnecessary radiation exposure or additional cost. The SAT and SM were segmented automatically based on a range of −190 to −30 and −29 to 150 Hounsfield units (HU), respectively 26. The tissue cross‐sectional areas (cm^2^) in selected regions were calculated automatically by the CT software. The SAT and muscle indices were calculated as the ratio of the selected area (cm^2^) divided by the height (m^2^). We further excluded 253 patients without proper alignment between the humeral heads and the T2 level, 303 patients with simulations performed more than 30 days before RT, and 211 patients with missing imaging data; ultimately, 881 patients were analyzed (Fig. 1). Since 2011, whole‐body positron emission tomography (PET)/CT scan has been used for initial tumor staging in HNC; 398 patients underwent PET/CT and simulation CT scans within 7 days of each other for SAT and SM measurements at the levels of the third lumbar vertebra (L3) and T2. All subjects provided informed consent to participate in the study.

### Definition of the study variables

SAT and SM indices were dichotomized into high and low groups based on their median values, with specific cutoffs for each sex. BMI was categorized into underweight (≤18.5 kg/m^2^), normal weight (18.5–24.99 kg/m^2^), overweight (25–29.99 kg/m^2^), and obese (≥30 kg/m^2^). To correct for different total radiation doses and fraction sizes, the equivalent dose in 2‐Gy fractions (EQD2) was used for analysis. T‐ and N‐stages were categorized into early versus late. The histories of risky oral habits (betel quid chewing, cigarette smoking, and alcohol drinking) were collected by means of a questionnaire on the date of first consultation with a radiation oncologist as previously described 4. Briefly, betel quid (no: never; yes: current or former chewer), alcohol (no: never; yes: current or former drinker), and smoking (no: never or smoked less than 100 cigarettes during the lifetime; yes: at least 100 cigarettes during the lifetime). The burden of comorbidity was dichotomized (yes vs. no) using the Charlson comorbidity index 4, 27. The Charlson index score was established by medical chart review and self‐reported by patients. In this study, the presence of a HNC (corresponding to a score of 6) was categorized as no.

### Statistical analysis

Patients were periodically followed up until death from any cause or until the cutoff date of the analysis, 1 September 2017. The primary outcome of interest was overall survival (OS). Secondary outcomes were locoregional control (LRC) and metastasis‐free survival (MFS). OS was defined as the interval between the dates of pathologically proven diagnosis and death from any cause. LRC was defined as the interval between diagnosis and locoregional recurrence, while MFS was defined as the interval between the dates of diagnosis and detection of DM. Two‐tailed *t*‐tests (for continuous variables) and Pearson chi‐square tests (for categorical variables) were used to assess the differences in clinical parameters between groups. Data were presented as mean (SD) and median (95% confidence interval [CI]). The cutoffs for SAT and SM in the T2 level indices were the median values in each of the sexes. Pearson's (*γ*) coefficients were calculated for correlation among each of body compositions at the level of T2 and L3. Survival curves were plotted by the Kaplan–Meier method and compared between groups with the log‐rank test. A multivariate Cox proportional hazards model was used to test significant predictors of OS/LRC/MFS. Hazard ratios (HRs) and corresponding 95% CIs are presented. All the patient‐, tumor‐, and treatment‐related variables were included as potential prognostic factors in multivariate analysis. Two‐tailed *P*‐values <0.05 were considered statistically significant.

## Results

### Patient characteristics

Patients were followed until death (*n* = 325) or censoring (the date last known to be alive; *n* = 563). The median follow‐up time for survivors was 4.68 years (range: 0.15–11.24 years). The demographic data of patients according to sex and body composition are shown in Tables 1 and 2. Mean age was 50.81 years (range, 19.3‐85.55 years). Among all patients, 82.7% (*n* = 729) of patients were male versus 17.3% (*n* = 152) were female. 50.4% (*n* = 444) had early T‐stage disease, and 49.6% (*n* = 437) had late‐stage disease. Approximately half of the cancers were nasopharyngeal carcinoma (NPC). The duration of RT ranged from 39 to 70 days (median, 52 days); the mean EQD_2 Gy_ was 72 Gy (range, 64–74 Gy). 86.9% (*n* = 766) were treated with platinum‐base chemotherapy. Female patients had higher rates of NPC and lower rates of hypopharyngeal carcinoma than males and were also more associated with early T‐ and N‐stage (*P* < 0.001 and *P* = 0.009, respectively). Unhealthy lifestyle habits including smoking (*P* < 0.001), betel quid chewing (*P* < 0.001), and alcohol consumption (*P* < 0.001) were significantly associated with male patients. Early T‐stage (*P* = 0.029) was more common than advanced T‐stage in patients with a high SAT index, while early T‐ (*P* = 0.014) and N‐stages (*P* = 0.04) were more common than advanced T‐ and N‐stages in the high SM index group. For 173 patients with whole‐body CT images, a positive correlation was seen between SAT index at the level T2 and SAT index at the level of L3 (*r* = 0.822; *P* < 0.001). SM indices were also positively correlated between T2 and L3 level (*r* = 0.63; *P* < 0.001).

**Table 1:** Patient distribution according to sex

	Female	Male	Overall	*P* value
Number	152 (17.3%)	729 (82.7%)	881 (100%)	
SAT index, (Median)	18.6 (0.93–88.09)	6.19 (0.21–40.48)	7.03 (0.21–88.09)	<0.001^b^
Mean (±SD), cm^2^/m^2^	20.04 ± 16.11	7.84 ± 6.14	9.95 ± 9.85	
SM index, (Median)	34.30 (14.78–73.05)	51.74 (8.03–89.06)	48.56 (8.03–89.06)	<0.001^b^
Mean (±SD), cm^2^/m^2^	35.32 ± 10.34	51.80 ± 13.48	48.96 ± 14.41	
BMI (kg/m^2^), Median	23.33 (17.10–33.17)	23.56 (13.87–37.30)	23.43 (13.87–37.30)	0.936^b^
Mean (±SD), cm^2^/m^2^	23.70 ± 3.66	23.73 ± 3.58	23.72 ± 3.59	
Underweight, *n* (%)	7 (4.6%)	50 (6.9%)	57 (6.5%)	0.591^1^
Normal, *n* (%)	95 (62.5%)	428 (58.7%)	523 (59.4%)	
Overweight, *n* (%)	41 (27.0%)	216 (29.6%)	257 (29.2%)	
Obese, *n* (%)	9 (5.9%)	35 (4.8%)	44 (5%)	
EQD_2 Gy_ (Median)	72 (64–74)	72 (64–74)	72 (64–74)	0.456^b^
Mean (±SD)	71.3 ± 1.45	71.2 ± 1.56	71.26 ± 1.51	
Treatment days (Median)	52 (44–69)	52 (39–70)	52 (39–70)	0.956^b^
Mean (±SD)	53.49 ± 4.47	53.47 ± 4.73	53.48 ± 4.60	
Age (years) Median	49.51 (19.30–85.55)	52.12 (26.97–85.22)	50.81 (19.30–85.55)	<0.001^b^
Mean (±SD)	49.55 ± 10.86	53.25 ± 11.61	51.40 ± 11.39	
Lesion, *n* (%)				
Nasopharynx	119 (78.3%)	323 (44.3%)	442 (50.2%)	<0.001^b^
Oral cavity	4 (2.6%)	41 (5.6%)	45 (5.1%)	
Oropharynx	23 (15.1%)	158 (21.7%)	181 (20.5%)	
Hypopharynx	1 (0.7%)	146 (20.0%)	147 (16.7%)	
Larynx	5 (3.3%)	61 (8.4%)	66 (7.5%)	
T‐Stage, *n* (%)				
T_2/1_	103 (67.8%)	341 (46.8%)	444 (50.4%)	<0.001^b^
T_4/3_	49 (32.2%)	388 (53.2%)	437 (49.6%)	
N‐Stage, *n* (%)				
N_1/0_	94 (61.8%)	366 (50.2%)	460 (52.2%)	0.009^a^
N_3/2_	58 (38.2%)	363 (49.8%)	421 (47.8%)	
Chemotherapy, *n* (%)				
No	26 (17.1%)	89 (12.2%)	115 (13.1%)	0.103^a^
Yes	126 (82.9%)	640 (87.8%)	766 (86.9%)	
PET study, *n* (%)				
No	20 (13.2%)	113 (15.5%)	133 (15.1%)	0.463^a^
Yes	132 (86.8%)	616 (84.5%)	748 (84.9%)	
Smoking, *n* (%)				
No	131 (86.2%)	160 (21.9%)	291 (33.0%)	<0.001^b^
Yes	21 (13.8%)	569 (78.1%)	590 (67.0%)	
Betel Quid, *n* (%)				
No	144 (94.7%)	368 (50.5%)	512 (58.1%)	<0.001^b^
Yes	8 (5.3%)	361 (49.5%)	369 (41.9%)	
Alcohol, *n* (%)				
No	134 (88.2%)	334 (45.8%)	468 (53.1%)	<0.001^b^
Yes	18 (11.8%)	395 (54.2%)	413 (46.9%)	
Comorbidity, *n* (%)				
No	97 (63.8%)	404 (55.4%)	501 (56.9%)	0.057^a^
Yes	55 (36.2%)	325 (44.6%)	380 (43.1%)	

SAT, subcutaneous fat tissue; SM, skeletal muscle; BMI, body mass index; EQD_2 Gy,_ equivalent dose in 2‐Gy fractions; Gy, gray; PET, positron emission tomography; Index: area/height/height.

a Chi‐square test.

b anova.

**Table 2:** Patient distribution according to body composition

	SAT index			SM index		
	Low	High	*P* value	Low	High	*P* value
Number	441	440		441	440	
SAT index, (Median)	–	–		5.93 (0.22–32.42)	8.69 (0.21–88.09)	<0.001^b^
Mean (±SD), cm^2^/m^2^	–	–		7.90 ± 6.61	11.99 ± 11.93	
SM index, (Median)	44.88 (14.78–89.03)	51.72 (8.03–89.06)	<0.001^b^	–	–	<0.001^b^
Mean (±SD), cm^2^/m^2^	46.31 ± 13.91	51.60 ± 14.43		–	–	
BMI (kg/m^2^), Median	21.43 (13.87–28.26)	25.61 (17.24–37.30)	<0.001^b^	21.93 (13.87–31.78)	25.12 (16.87–37.30)	<0.001^b^
Mean (±SD), cm^2^/m^2^	21.48 ± 2.52	25.97 ± 3.07		22.12 ± 3.02	.25.33 ± 3.40	
Underweight, *n* (%)	55 (12.5%)	2 (0.5%)	<0.001^a^	54 (12.2%)	3 (0.7%)	<0.001^a^
Normal, *n* (%)	345 (78.2%)	178 (40.5%)		314 (71.2%)	209 (47.5%)	
Overweight, *n* (%)	41 (9.3%)	216 (49.1%)		69 (15.6%)	188 (42.7%)	
Obese, *n* (%)	0 (0%)	44 (10.0%)		4 (0.9%)	40 (9.1%)	
EQD_2 Gy_ (Median)	72 (64–74)	72 (64–74)	0.456^b^	72 (64–74)	72 (64–74)	0.997^b^
Mean (±SD)	71.3 ± 1.45	71.2 ± 1.56		71.31 ± 1.59	71.21 ± 1.42	
Treatment days (Median)	52 (44–69)	52 (39–70)	0.956^b^	52 (44–70)	52 (39–70)	0.063^b^
Mean (±SD)	53.49 ± 4.47	53.47 ± 4.73		53.44 ± 4.59	53.52 ± 4.62	
Age (years) Median	49.5 (19.3–85.56)	52.1 (27–85.2)	<0.001^b^	51.4 (19.3–85.2)	50.572 (21.7–85.6)	0.135^b^
Mean (±SD)	49.5 ± 10.8	53.35 ± 11.6		52.0 ± 12.0	50.8 ± 10.8	
Lesion, *n* (%)			0.061^a^			<0.001^a^
Nasopharynx	211 (47.8%)	231 (52.5%)		187 (42.4%)	255 (58.0%)	
Oral cavity	24 (5.4%)	21 (4.8%)		31 (7.0%)	14 (3.2%)	
Oropharynx	91 (20.6%)	90 (20.5%)		100 (22.7%)	81 (18.4%)	
Hypopharynx	88 (20.0%)	59 (13.4%)		89 (20.2%)	58 (13.2%)	
Larynx	27 (6.1%)	39 (8.9%)		34 (7.7%)	32 (7.3%)	
Sex, *n* (%)			0.988^a^			0.988^a^
Female	76 (17.2%)	76 (17.3%)		76 (17.2%)	76 (17.3%)	
Male	365 (82.8%)	364 (82.7%)		365 (82.8%)	364 (82.7%)	
T‐Stage, *n* (%)			0.029^a^			0.014^a^
T_2/1_	206 (46.7%)	238 (54.1%)		204 (46.3%)	240 (54.5%)	
T_4/3_	235 (53.3%)	202 (45.9%)		237 (53.7%)	200 (45.5%)	
N‐Stage, *n* (%)			0.212^a^			0.040^a^
N_1/0_	221 (50.1%)	239 (54.3%)		215 (48.8%)	245 (55.7%)	
N_3/2_	220 (49.9%)	201 (45.7%)		226 (51.2%)	195 (44.3%)	
Chemotherapy, *n* (%)			0.774^a^			0.626^a^
No	59 (13.4%)	56 (12.7%)		60 (13.6%)	55 (12.5%)	
Yes	382 (86.6%)	384 (87.3%)		381 (86.4%)	385 (87.5%)	
PET study, *n* (%)			0.628^a^			0.767^a^
No	64 (14.5%)	69 (15.7%)		65 (14.7%)	68 (15.5%)	
Yes	377 (85.5%)	371 (84.3%)		376 (85.3%)	372 (84.5%)	
Smoking, *n* (%)			0.070^a^			0.811^a^
No	133 (30.2%)	158 (35.9%)		144 (32.7%)	147 (33.4%)	
Yes	308 (67.3%)	282 (64.1%)		297 (67.3%)	293 (66.6%)	
Betel Quid, *n* (%)			0.653^a^			0.185^a^
No	253 (57.4%)	259 (58.9%)		266 (60.3%)	246 (55.9%)	
Yes	188 (42.6%)	181 (41.1%)		175 (39.7%)	194 (44.1%)	
Alcohol, *n* (%)			0.760^a^			0.760^a^
No	232 (52.6%)	236 (53.6%)		323 (52.6%)	236 (53.6%)	
Yes	209 (47.4%)	204 (46.4%)		209 (47.4%)	204 (46.4%)	
Comorbidity, *n* (%)			<0.001^b^			0.264
No	291 (66.0%)	210 (47.7%)		259 (58.7%)	242 (55.0%)	
Yes	150 (34.0%)	230 (52.3%)		182 (41.3%)	198 (45.0%)	

SAT, subcutaneous fat tissue; SM, skeletal muscle; BMI, body mass index; EQD_2 Gy,_ equivalent dose in 2‐Gy fractions; Gy, gray; PET, positron emission tomography; Index: area/height/height.

a Chi‐square test.

b anova.

### Comparison of SAT index, SM index, and BMI by sex

Image data from CT scans showed significant differences in both SAT and SM indices by sex (Table 1). Female patients had a significantly higher SAT index than males (mean, 20.03 ± 16.11 cm^2^/m^2^ of female vs. 7.84 ± 6.14 cm^2^/m^2^ of male; *P* < 0.001), whereas an opposing significant sex difference in SM index was observed (mean, 35.32 ± 10.34 cm^2^/m^2^ of female versus 51.8 ± 13.48 cm^2^/m^2^ of male; *P* < 0.001). There is no difference in BMI between female (mean, 23.7 ± 3.66 kg/m^2^) and male (mean, 23.73 ± 3.58 kg/m^2^) (*P* = 0.936). A total of 57 patients (6.5%) were underweight, 523 (59.4%) were normal BMI, 257 (29.2%) were overweight, and 44 (5%) were obese. The SAT index and SM index were significantly correlated with BMI in entire population (SAT index: *r* = 0.63, *P* < 0.001; SM index: *r* = 0.48, *P* < 0.001), women (SAT index: *r* = 0.859, *P* < 0.001; SM index: *r* = 0.508, *P* < 0.001), and men (SAT index: *r* = 0.749, *P* < 0.001; SM index: *r* = 0.508, *P* < 0.001).

### Locoregional, distant, and survival outcomes

**Figure 2:**
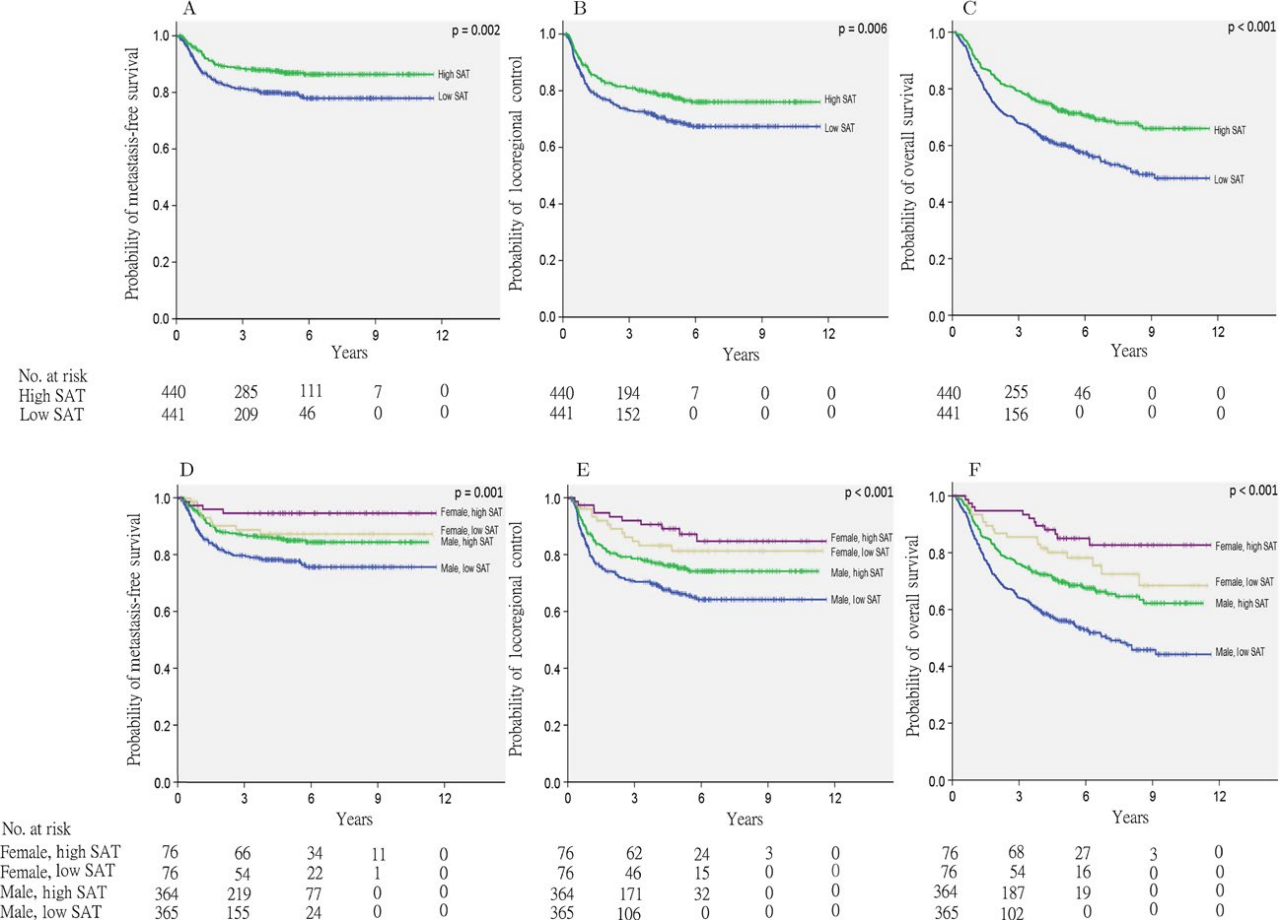
Kaplan–Meier estimates of metastasis‐free survival (A), locoregional control (B), and overall survival (C), according to subcutaneous adipose tissue (SAT) status. Kaplan–Meier estimates of metastasis‐free survival (D), locoregional control (E), and overall survival (F), according to SAT status and sex.

Of the 881 patients, 219 (24.9%) developed locoregional recurrence and 138 (15.7%) developed DM. The median MFS and LRC times were not reached. The actuarial 5‐ and 10‐year OS rates were 66.4% and 57.6%, respectively, with a median OS of 4.68 years. Table 3 shows the 5‐ and 10‐year MFS, LRC, and OS of patients according to their SAT and SM indices and sex. High SAT before treatment was significantly associated with superior MFS (HR: 0.585, 95% CI: 0.416–0.824, *P* = 0.002), LRC (HR: 0.688, 95% CI: 0.526–0.899, *P* = 0.006), and OS (HR: 0.608, 95% CI: 0.487–0.759, *P* < 0.001) on univariate analysis (Table 4). High SAT leads to improvement in 5‐year LRC from 69% to 77.2% and 5‐year MFS from 79.1% to 86.5%. OS was increased from 60.3% to 72.2% at 5 years, and the difference (48.5% to 66.7%) became more significant at 10 years in patients with high SAT. High SM before treatment was significantly associated with superior LRC (HR: 0.684, 95% CI: 0.523–0.894, *P* = 0.005) and OS (HR: 0.60, 95% CI: 0.48–0.749, *P* < 0.001), but not MFS (HR: 0.80, 95% CI: 0.573–1.119, *P* = 0.192) on univariate analysis. Figure 2 shows the MFS, LRC, and OS according to the SAT group.

**Table 3:** Outcomes of patients according to body composition and sex

	SAT index		SM index				
	Low (%)^a^	High (%)^b^	Low (%)^b^	High (%)^2^	Male (%)	Female (%)	Overall (%)
Metastasis‐free survival							
5 years	79.2	86.7	81.3	84.6	81.3	91.1	83.0
10 years	77.7	86.3	80.9	83.4	80.0	91.1	82.1
Locoregional control							
5 years	69.0	77.3	68.7	77.5	70.8	84.6	73.4
10 years	66.8	75.6	66.8	75.6	68.9	82.6	71.4
Overall survival							
5 years	60.3	72.2	59.2	73.2	62.8	83.0	66.4
10 years	48.5	66.7	50.2	64.8	53.3	77.6	57.6

SAT, subcutaneous fat tissue; SM, skeletal muscle.

a Low SAT defined as SAT index <19.49 cm^2^/m^2^ for women and <7.24 cm^2^/m^2^ for men, high SAT defined as SAT index ≥ 19.49 cm^2^/m^2^ for women and ≥7.24 cm^2^/m^2^ for men.

b Low SM defined as SM index <34.3 cm^2^/m^2^ for women and < 51.74 cm^2^/m^2^ for men, high SM defined as SM index ≥ 34.3 cm^2^/m^2^ for women and ≥ 51.74 cm^2^/m^2^ for men.

**Table 4:** Univariate analyses of metastasis‐free survival, locoregional control, and overall survival

	MFS		LRC		OS	
Covariate	HR (95% CI)	*P* value	HR (95% CI)	*P* value	HR (95% CI)	*P* value
T‐Stage (T_4/3_ vs. T_2/1_)	2.488 (1.745–3.547)	<0.001	1.646 (1.260–2.152)	<0.001	2.691 (2.133–3.393)	<0.001
N‐Stage (N_3/2_ vs. N_1/0_)	3.617 (2.480–5.274)	<0.001	2.107 (1.605–2.764)	<0.001	2.388 (1.904–2.995)	<0.001
Lesion		<0.001		<0.001		<0.001
Oral cavity vs. NPC)	2.965 (1.511–5.816)	0.002	5.500 (3.396–8.907)	<0.001	8.215 (5.526–12.213)	<0.001
Oropharynx vs. NPC)	1.683 (1.103–2.568)	0.016	2.052 (1.456–2.892)	<0.001	2.898 (2.162–3.885)	<0.001
Hypopharynx vs. NPC)	2.188 (1.421–3.370)	<0.001	2.270 (1.573–3.275)	<0.001	3.992 (2.973–5.360)	<0.001
Larynx vs. NPC)	0.351 (0.110–1.119)	0.077	1.527 (0.906–2.573)	0.112	1.995 (1.294–3.078)	0.002
Sex (male vs. female)	2.282 (1.289–4.042)	0.005	2.130 (1.382–3.281)	0.001	2.383 (1.646–3.452)	<0.001
Smoking (yes vs. no)	1.722 (1.169–2.538)	0.006	2.636 (1.876–3.704)	<0.001	2.443 (1.860–3.209)	<0.001
Betel quid (yes vs. no)	1.650 (1.182–2.306)	0.003	2.027 (1.553–2.644)	<0.001	2.209 (1.773–2.751)	<0.001
Alcohol (yes vs. no)	1.501 (1.074–2.099)	0.017	2.040 (1.555–2.676)	<0.001	1.992 (1.594–2.488)	<0.001
Chemotherapy (yes vs. no)	1.601 (0.886–2.895)	0.119	1.383 (0.890–2.148)	0.150	1.144 (0.816–1.605)	0.436
Age (years)^a^	0.990 (0.975–1.005)	0.194	0.998 (0.987–1.010)	0.776	1.024 (1.014–1.033)	<0.001
Treatment days^a^	1.030 (0.995–1.066)	0.096	1.026 (0.998–1.054)	0.069	1.042 (1.020–1.065)	<0.001
EQD_2 Gy_ a	1.084 (0.954–1.231)	0.215	1.040 (0.946–1.144)	0.421	1.062 (0.980–1.150)	0.143
PET study (yes vs. no)	1.066 (0.657–1.731)	0.795	0.760 (0.538–1.075)	0.120	0.766 (0.578–1.014)	0.063
Comorbidity (yes vs. no)	0.784 (0.555–1.108)	0.168	0.997 (0.763–1.303)	0.982	1.215 (0.977–1.511)	0.081
SAT index (high vs. low)	0.585 (0.416–0.824)	0.002	0.688 (0.526–0.899)	0.006	0.608 (0.487–0.759)	<0.001
SM index (high vs. low)	0.800 (0.573–1.119)	0.192	0.684 (0.523–0.894)	0.005	0.600 (0.480–0.749)	<0.001

MFS, metastasis‐free survival; LRC, locoregional control; OS, overall survival; HR, hazard ratio; CI, confidence interval; NPC, nasopharyngeal carcinoma; EQD2 Gy, equivalent dose in 2‐Gy fractions; Gy, gray; PET, positron emission tomography; SAT, subcutaneous fat tissue; SM, skeletal muscle.

a Continuous variable.

### Prognostic study

As shown in Table 4, the following variables were all identified as significant prognostic factors for longer LRC on univariate analysis: high SAT index, high SM index, early T‐stage, early N‐stage, NPC or laryngeal carcinoma, female sex, no smoking history, no betel quid chewing, and no alcohol consumption. The following variables were identified as prognostic factors for longer MFS: high SAT index, early T‐stage, early N‐stage, NPC or laryngeal carcinoma, female sex, no smoking history, no betel quid chewing, and no alcohol drinking. The following variables were identified as prognostic factors for longer OS: high SAT index, high SA index, early T‐stage, early N‐stage, NPC or laryngeal carcinoma, female sex, no smoking history, no betel quid chewing, no alcohol consumption, younger age, shorter treatment duration, no comorbidity, and undergoing PET imaging for staging.

Patients were further grouped according to sex and body composition (SAT and SM indices). Increased MSF was observed in women with high SAT (HR: 0.207, 95% CI: 0.076–0.567; *P* = 0.002) and low SAT (HR: 0.492, 95% CI: 0.247–0.983; *P* = 0.045), as well as in men with high SAT (HR: 0.601, 95% CI: 0.420–0.860, *P* = 0.005) compared to men with low SAT (HR: 1.00) (Fig. 2D). The differences in MFS between women with high versus low SAT (HR: 0.421: 95% CI: 0.130–1.366, *P* = 0.15) or between women with low SAT and men with high SAT (HR: 1.22, 95% CI: 0.600–2.482, *P* = 0. 582) were not significant. However, women with high SAT had a significantly longer MFS than men with high SAT (HR: 0.343; 95% CI: 0.124–0.950, *P* = 0.039). LRC was significantly better in women with high SAT (HR: 0.358, 95% CI: 0.173–0.741, *P* = 0.006), women with low SAT (HR: 0.476, 95% CI: 0.247–0.918; *P* = 0.027), and men with high SAT (HR: 0.719, 95% CI, 0.521–0.992, *P* = 0.044), but no differences were observed between women with high versus low SATs (HR: 0.752, 95% CI: 0.297–1.906; *P* = 0.548) or between women with low SAT and men with high SAT (HR: 1.508, 95% CI: 0.776–2.931; *P* = 0.225) (Fig. 2E). Women with high SAT (HR: 0.212, 95% CI: 0.112–0.401, *P* = 0.0001), women with low SAT (HR: 0.408, 95% CI: 0.251–0.663, *P* = 0.0001), and men with high SAT (HR: 0.612, 95% CI: 0.484–0.774; *P* = 0.0001) showed longer OS compared to men with low SAT (HR: 1.00) (Fig. 2F). No significant difference in OS was observed between women with low SAT and men with high SAT (HR: 1.500, 95% CI: 0.914–2.464; *P* = 0.109). Women with high SAT showed longer OS than those with low SAT, although not significantly so (HR: 0.519, 95% CI: 0.240–1.125, *P* = 0.097).

Multivariate analysis of significant variables determined by univariate analysis identified three, five, and six prognostic factors that independently impacted on MFS, LRC, and OS (Table 5). High SAT retained a positive outcome on MFS, LRC, and OS with a HR of 0.65 (95% CI: 0.459–0.920, *P* = 0.015), 0.758 (95% CI: 0.577–0.997, *P* = 0.047), and 0.604 (95% CI: 0.478–0.762, *P* = <0.001). Early N‐stage was the other significant predictor for all the three outcomes: MFS (HR: 2.913, 95% CI: 19.78–4.290; *P* < 0.001), LRC (HR: 1.75, 95% CI: 1.321–2.318; *P* < 0.001), and OS (HR: 1.938, 95% CI: 1.526–2.461; *P* < 0.001). Sex and SM index lost their significance in the multivariate model.

**Table 5:** Multivariate analyses of metastasis‐free survival, locoregional control, and overall survival by body composition

	MFS		LRC		OS	
Covariate	HR (95% CI)	*P* value	HR (95% CI)	*P* value	HR (95% CI)	*P* value
T‐Stage (T_4/3_ vs. T_2/1_)	1.804 (1.247–2.608)	0.002	1.172 (0.885–1.552)	0.268	1.719 (1.346–2.195)	<0.001
N‐Stage (N_3/2_ vs. N_1/0_)	2.913 (1.978–4.290)	<0.001	1.750 (1.321–2.318)	<0.001	1.938 (1.526–2.461)	<0.001
Lesion (others vs. NPC)	1.145 (0.779–1.683)	0.055	1.441 (1.061–1.957)	<0.001	1.854 (1.405–2.447)	<0.001
Sex (male vs. female)	1.707 (0.894–3.258)	0.105	1.092 (0.659–1.808)	0.733	1.150 (0.749–1.768)	0.523
Smoking (yes vs. no)	0.990 (0.596–1.646)	0.970	1.669 (1.072–2.600)	0.023	1.266 (0.878–1.823)	0.206
Betel quid (yes vs. no)	0.996 (0.654–1.516)	0.984	1.103 (0.797–1.527)	0.555	1.206 (0.913–1.593)	0.188
Alcohol (yes vs. no)	1.106 (0.749–1.634)	0.612	1.376 (1.009–1.877)	0.044	1.271 (0.983–1.642)	0.067
Age (years)^a^					1.031 (1.019–1.043)	<0.001
Treatment days^a^					1.026 (1.004–1.049)	0.021
SAT index (high vs. low)	0.650 (0.459–0.920)	0.015	0.758 (0.577–0.997)	0.047	0.604 (0.478–0.762)	<0.001
SM index (high vs. low)			0.777 (0.588–1.027)	0.077	0.805 (0.639–1.015)	0.066

MFS, metastasis‐free survival; LRC, locoregional control; OS, overall survival; HR, hazard ratio; CI, confidence interval; NPC, nasopharyngeal carcinoma; EQD2 Gy, equivalent dose in 2‐Gy fractions; Gy, gray; PET, positron emission tomography; SAT, subcutaneous fat tissue; SM, skeletal muscle.

a Continuous variable.

## Discussion

To the best of our knowledge, ours is the largest study to investigate the impact of subcutaneous adiposity on outcomes in patients with pathologically proven HNC. We demonstrated that the SAT index obtained from a single pretreatment CT image slice of tissue can be used to predict outcomes in patients with HNCs. In our previous study of 1,562 patients with HNC, the risk of death was markedly lower among female patients. Studies in other countries have also shown that HNC cancer mortality is much higher in men than in women 23, 28, 29, 30, 31, 32. Several explanations for this have been proposed including the antioxidant effect of estrogen 33, regulation of innate and adaptive immunity by sex hormones 34, and gene expression differences 35. Other factors implicated an advantage in cancer surviving among females include susceptibility to carcinogens 36, body mass index 37, healthier behavior, and higher medical care service utilization 38. In our study, significantly higher LRC, OS, and MFS rates were observed in women on univariate but not multivariate analyses. Although the sexes were similarly distributed across BMI ranges, women had significantly higher subcutaneous fat indices than men. The volume of subcutaneous adipose tissue is not necessarily reflected by BMI 39.

In a systemic review, den Hollander et al. 40 found that higher BMI is associated with better OS, lower cancer‐related death, and fewer locoregional and distant failures. Moreover, Grossberg et al. 41 reported that BMI and SM depletion can predict the outcomes of patients with head and neck squamous cell carcinoma independently; they showed that BMI was the strongest performing factor, followed by postradiation SM depletion. However, they did not analyze the impact of adiposity loss, which can occur more rapidly than the reduction in lean muscle during cachexia 42. One of the main functions of adipose tissue is the regulation of whole‐body energy homeostasis. Patients with larger volumes of subcutaneous tissue loss may experience considerable energy depletion owing to cancer, leading to poor outcomes. In our study, patients with above‐median SAT indices in both sexes had significantly better outcomes.

Tumor cells exhibit increased adipose tissue lipolysis for obtaining fatty acid to support their growth and proliferation; robust lipolysis is a significant factor leading to cachexia in patients with cancer 43, 44. Adipose tissue depletion precedes muscle wasting in patients with cancer, and this phenomenon can be occurred before alternation in food intake and be accelerated at the disease progression 12. Batista et al. found that lipokines that disrupt lipid metabolism and increase lipolysis, such as interleukin (IL)‐6, adiponectin, tumor necrosis factor alpha, and IL‐10, are upregulated in the plasma of cancer patients with cachexia; lipokine mRNA is also upregulated in patients’ subcutaneous tissues 44. Adipose tissue atrophy during disease progression involves both increased adipose catabolism and extracellular matrix (ECM) remodeling in adipose tissue; moreover, the disruption of SAT may occur before the manifestation of clinical symptoms 45. Another study of Japanese patients with multiple myeloma found that a low baseline SAT volume predicted inferior overall survival, with evidence of hypercatabolism in tumors being associated with SAT loss 46. Hence, changes in SAT may predict cancer aggressiveness, with SAT depletion being related to poorer outcomes. Our results are consistent with Ebadi et al. 47 who found that a high subcutaneous adiposity was significantly associated with longer survival in 1762 patients with cancer. Although lack of treatment data and the majority of patients were in stage IV in their study, results may not apply to the patients in earlier stage of cancer. In our study, all patients were treated with curative intent and half of patients were in the earlier cancer stage. Besides, our data demonstrate that subcutaneous adiposity is an independent factor not only for overall survival but also for local control and distant metastasis.

Consistent with Grossberg et al. 42, we found no relationship between preradiation SM and survival or locoregional failure and DM. In their study, BMI and postradiation SM depletion were independent predictive factors of OS. Patients with BMIs <25 kg/m^2^ had higher mortality rates than obese patients, while patients with SM depletion after completing at least 8 weeks of RT had higher mortality rates than those without SM depletion. In our study, the BMI data and CT images were collected within two weeks and 30 days before RT, respectively, which are much shorter intervals than those of Grossberg et al. (60 days). Our results ought to be more representative of the actual nutrition status of patients at the time of treatment.

The study is limited by several factors. First, our retrospective design restricted which covariates could be analyzed. Most clinical factors were determined based on medical chart reviews, and it is possible that there was some degree of misclassification. Secondary, adipose tissue index cutoff points have yet to be defined in both sexes. Also, BMI and body composition cutoff points have not defined for the Asian population. The underlying mechanism of how adipose tissue affects outcomes in patients with HNC could not be explained by the present study. Molecular factors and biomarkers should be used in the further research. Additionally, body composition in our study was determined using the T2 CT section instead of L3, although the latter was shown to more representative 48. Owing to the retrospective nature of the study, whole‐body PET/CT scanning was not routinely performed in patients with HNC before 2012. We overcame this limitation by evaluating the correlation in body composition between the T2 and L3 levels in 173 patients who underwent whole‐body PET/CT post‐2012. Both SAT and SM were significantly correlated between the T2 and L3 levels, indicating our data's reliability. Lack of visceral adipose tissue data was also a limitation. It is still controversial whether visceral adiposity associated with cancer survival 49. Our unpublished data showed that higher visceral adipose tissue is at greater risk of local recurrence and mortality in patients with head and neck cancer.

In conclusion, this largest study of its kind demonstrated a relationship between pretreatment SAT and outcomes of patients with HNC; patients with higher SAT indices before treatment experience better LRC, MFS, and OS rates. In contrast, SM appears to have no significant influence on LRC, MFS, or OS. We therefore recommend that pretreatment SAT is used for risk stratification in patients with HNC.

## Conflict of Interest

None declared.
